# *Cronobacter sakazakii* induced sepsis-associated arrhythmias through its outer membrane vesicles

**DOI:** 10.1016/j.isci.2024.110572

**Published:** 2024-07-25

**Authors:** Zhi-ping Fu, Shuang Lee, Rui-yao Wang, Yu-qing Wang

**Affiliations:** 1 Collage of Pharmacology, North China University of Science and Technology, Tangshan 063200, China

**Keywords:** Biological sciences, Microbiology, Pathophysiology, Physiology

## Abstract

Sepsis-induced arrhythmia, linked to sudden cardiac death, is associated with gut microbiota, though the exact relationship is unclear. This study aimed to elucidate the relationship between *Cronobacter sakazakii* (*C. sakazakii*) and arrhythmia. The relative abundance of *C. sakazakii* was increased in cecal ligation and puncture (CLP)-induced septic mice. Live *C. sakazakii*, supernatant, and outer membrane vesicles (OMVs) resulted in premature ventricular beat (PVB), sinus arrhythmia (SA), and increased arrhythmia and mortality in sepsis model through dysregulated ion channel proteins. Moreover, short-chain fatty acids (SCFAs) showed antibacterial effects *in vitro*. We confirmed sodium acetate (C2) and sodium butyrate (C4) protect from *C. sakazakii*-induced arrhythmia, and C2 and C4 protected from septic arrhythmia by activating free fatty acid receptor 2 and 3 (FFAR2 and FFAR3) in mice. These findings point to how *C. sakazakii*’s OMVs trigger arrhythmia, and SCFAs may be a treatment for septic arrhythmia.

## Highlights

• Septic arrhythmia is associated with intestinal *Cronobacter sakazakii* abundance
• *C. sakazakii*-derived OMVs promote arrhythmia by damaging ion channel protein
• SCFAs showed antibacterial activities on *C. sakazakii*
• SCFAs reduced arrhythmia by activating cardiac FFAR2 and 3 in sepsis mice

## Graphical abstract

**Figure:**
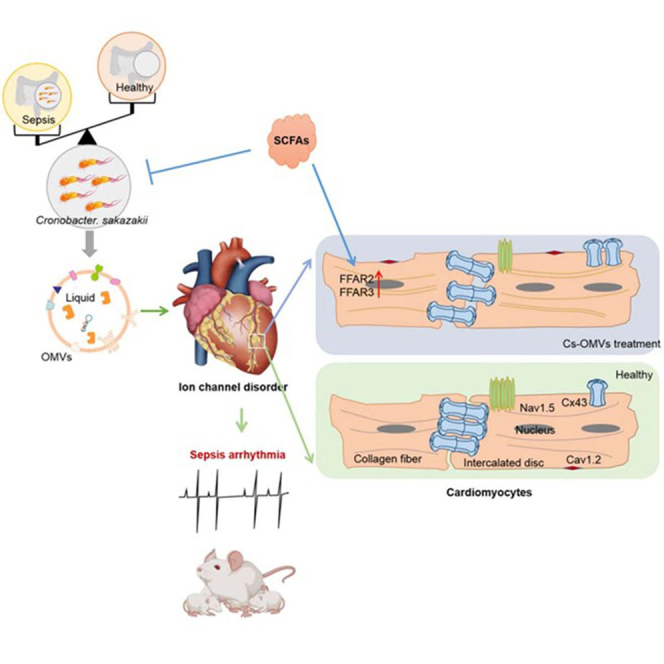

## Introduction

Sepsis is related to intrarenal and systemic inflammation and cardiac dysfunction, resulting in life-threatening cardiomyopathy and heart failure and high morbidity and mortality.^1^ Millions of patients are diagnosed with sepsis annually, leading to 30%–40% mortality rate. Sepsis-related arrhythmia is associated with higher mortality, and an adverse prognosis in sepsis-related arrhythmia indicated a lack of clinically therapeutic targets and treatments.^2^ Therefore, research on the pathogenesis of sepsis-related arrhythmia to find effective treatments are necessary. The gut microbiota serves as a crucial regulator in the gut and extra-gut organs, influencing a range of physiological processes and suggesting a bidirectional crosstalk between the intestinal flora and distal extra-intestinal organs and tissues.^3^ Previous studies have reported that intestinal flora in the gastrointestinal tract remotely modulates the pathophysiology of various disease processes and plays a role in sepsis-related arrhythmia. Gut microbiota dysbiosis has been shown to enhance arrhythmia, myocardial damage, and mortality in sepsis. In contrast, septic mice with an intestinal germ-free status established by an antibiotic cocktail exhibited lower cardiac remodeling, cardiometabolic risk, bacterial load, and mortality rates, suggesting that the intestinal microbiota serves as a crucial factor in sepsis-related arrhythmia and myocardial damage.^4^^,^^5^^,^^6^^,^^7^^,^^8^ Gut microbiota regulates septic cardiomyopathy and is involved in sepsis progression, which may be a potential risk factor in the development of sepsis-related arrhythmia.

A study showed that the 28-day mortality rate among patients with septic shock in the intensive care unit (ICU) was 43.6%.^9^ The occurrence rate of arrhythmias can also be attributed to many pathogenic factors. One of them is exotoxins, such as streptolysin O or pneumolysin, which possess cardiotoxic potential and can lead to septic cardiomyopathy and arrhythmias.^10^ Endotoxins, such as lipopolysaccharide (LPS) derived from the cell walls of gut microbiota bacteria, may contribute to the development of arrhythmia, possibly by the activation of the NLRP3-inflammasome and Toll-like receptors.^11^^,^^12^ Inflammatory mediators and mitochondrial dysfunction also contribute to the development of cardiac arrhythmias. A recent clinical study has shown that *Cronobacter* or its phage may have significant prognostic value for ventricular arrhythmias in patients with sepsis-induced cardiomyopathy.^13^ *Cronobacter* is an important foodborne bacterial pathogen related to fatal infections, including meningitis, necrotizing enterocolitis, and septicemia in newborn infants, which exhibits extreme invasiveness and causes damage to brain tissue.^14^ Meanwhile, *C. sakazakii-derived* outer membrane vesicles (OMVs) have been shown to contribute to the pathogenesis process by transporting toxic substances produced by the gut microbiota across the intestinal barrier, resulting in abnormal proliferative and proinflammatory responses.^15^ *C. sakazakii-derived* outer membrane vesicles (OMVs) possess multiple functions, including the ability to acquire necessary nutrients and deliver disease factors. Through the transmission of *C. sakazakii-derived* OMVs, pathogens can be efficiently concentrated and delivered to host cells while avoiding damage from external factors. According to recent reports, the OMVs produced by *C. sakazakii* play a bridging role between pathogens and host cells, mediating the delivery of toxins and other pathogens to host cells. These OMVs are rich in membrane lipids, peptidoglycan, and proteins derived from the bacterial cell wall of the parent bacteria, providing bacteria with abundant biological information. Additionally, *C. sakazakii-derived* OMVs promote the activation of interleukin-8 in host cells by delivering proinflammatory substances such as lipopolysaccharides or bacterial cell wall virulence proteins, triggering a series of immune responses.^15^ However, the critical impact of Cronobacter on sepsis-induced arrhythmogenesis, as well as the underlying mechanisms, remains largely unknown.

This study reveals a new mechanism that elucidates the effects of *C. sakazakii*-derived OMVs on arrhythmogenesis through preclinical sepsis animal models. Our study found that the development of sepsis-related arrhythmia is link to gut *C. sakazakii* abundance in cecal ligation and puncture (CLP) mice, indicating a potential connection between *C. sakazakii* and arrhythmia caused by sepsis. Then, we identified the crucial role of *C. sakazakii*-derived OMVs in sepsis-related arrhythmia by the induction of cardiac ion channel protein dysregulation. Subsequent analyses revealed that sodium acetate and sodium butyrate exhibited good antibacterial activities on *C. sakazakii*- and sepsis-induced arrhythmia. We expanded the understanding of pathophysiological function of *C. sakazakii*-derived OMVs and provided a new possible therapeutic method for treating sepsis-related arrhythmia.

## Result

### Intestinal *C. sakazakii* participated in sepsis progression and arrhythmia in CLP mice

**Figure 1:**
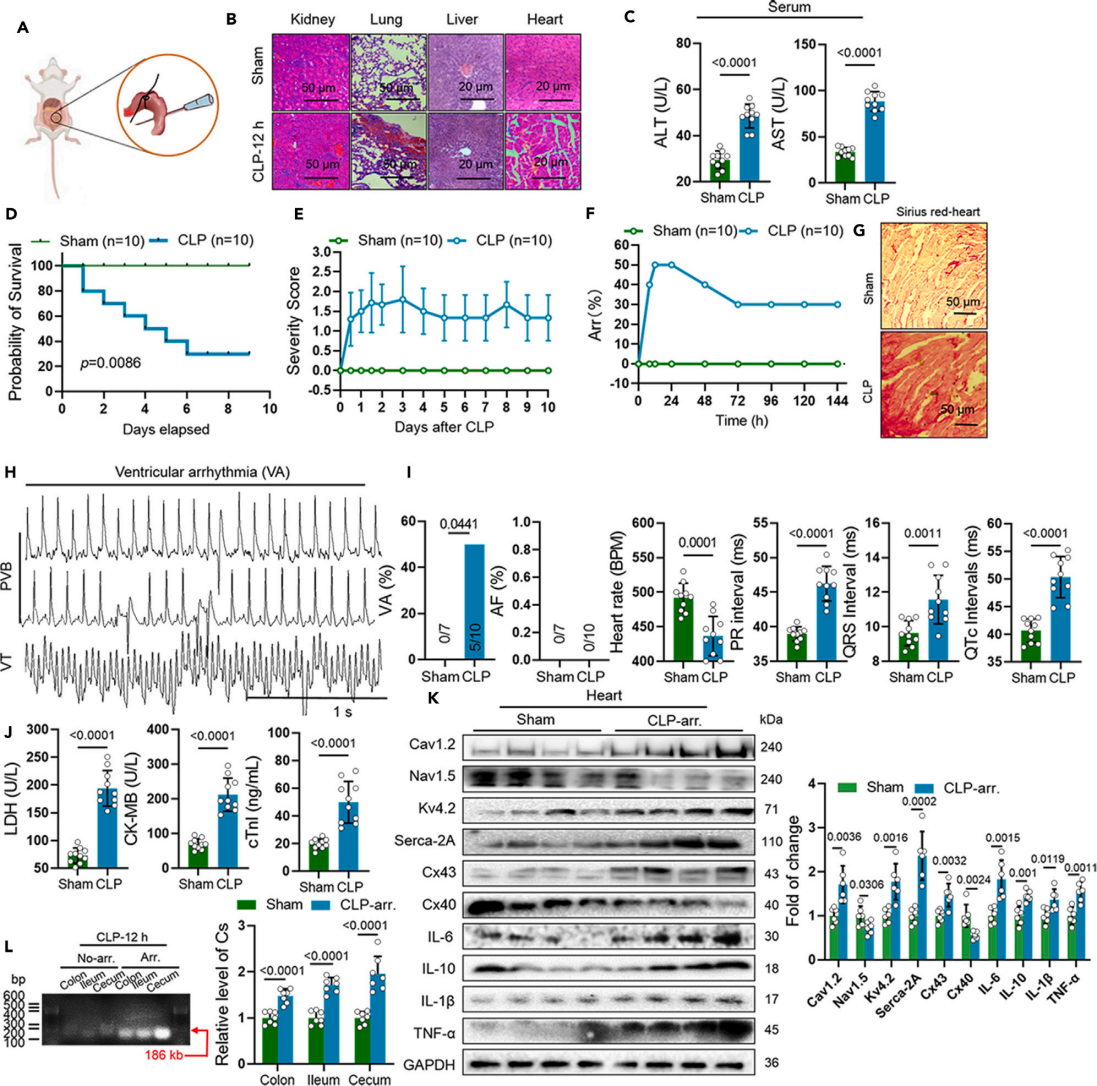
Intestinal *C. sakazakii* participated in sepsis progression and arrhythmia in CLP mice (A) Experimental design for sepsis mice model established by cecal ligation and puncture (CLP) surgery. (B) Representative hematoxylin and eosin (H&E) staining images of kidney, lung, liver, and heart in each group. (C) Serum alanine aminotransferase (ALT) and aspartate aminotransferase (AST) levels were quantified using commercial assay kits. Data are presented as the mean ± SD. Data analyzed by unpaired two-tailed Student’s t test. (D and E) Probability of survival and sepsis severity score were calculated for each group. Data are presented as the mean ± SD. (F) Concurrence of arrhythmia in mice after cecal ligation and puncture (CLP) challenge for 10 days. The peak occurrence of arrhythmia in septic mice was 50% in 12 h. (G) Ventricular fibrosis in sepsis mice after CLP challenge for 12 h was measured by Sirius red staining. (H) Sample surface electrocardiogram (ECG) traces of two different types of arrhythmic events: premature ventricular beats (PVB) and ventricular tachycardia (VT). Scale bar: 1 s. (I) Summary of the occurrence of ventricular arrhythmia (VA) and atrial fibrillation (AF) in sham and CLP mice. And differences in heart rates, PR intervals, QRS intervals, and QTc intervals were showed among each group. Numbers in parentheses indicate the number of mice that occurred VA followed by sham or CLP surgery (*n* = 10/per group). Data are presented as the mean ± SD. Data analyzed by unpaired two tailed Student’s t test. (J) Cardiac lactate dehydrogenase (LDH), creatine kinase-MB (CK-MB), and cardiac troponin I (cTnI) levels were quantified using commercial assay kits (*n* = 10/per group). Data are presented as the mean ± SD. Data analyzed by unpaired two-tailed Student’s t test. (K) Representative western blot and quantification of Cx43, Cx40, Nav1.5, Cav1.2, Serca-2A, Kv4.2, IL-6, IL-10, IL-1β, TNF-α, and GAPDH in ventricle of sham and CLP mice (*n* = 6/per group). GAPDH was used for internal normalization. Data are presented as the mean ± SD. Data analyzed by unpaired two-tailed Student’s t test. (L) Relative levels of *C*. *sakazakii* extracted from colon, ileum, and cecum content of no arrhythmia and arrhythmia septic mice were evaluated by PCR (*n* = 7/per group). Data are presented as the mean ± SD. Data analyzed by unpaired two-tailed Student’s t test. Cx43, Connexin 43; Cx40, Connexin 40; ISO, isoproterenol; IL-6, interleukin-6; TNF-α, tumor necrosis factor alpha; TGF-β, transforming growth factor β; Arr, arrhythmia; Cs, *C*. *sakazakii*.

Sepsis is defined as a systemic inflammation syndrome contributing to global injury of multiple organs, among which the heart is the one to be impacted.^1^^,^^2^ To investigate the function of *C. sakazakii* (Cs) arrhythmia caused by sepsis, a CD-1 mouse (8 weeks old) model of sepsis was established using CLP surgery (Figure 1A). Pathological changes were observed through the use of H&E staining, which showed normal live, lung, kidney, and heart structure in sham-operated mice, and damaged and disorder structure accompanied by inflammatory cell infiltration in CLP mice (Figure 1B). In addition, biochemistry analyses of serum showed significantly elevated levels of ALT and AST in CLP septic mice when compared to PBS-treated mice in 12 h after CLP surgery (Figure 1C). Furthermore, we utilized a severity score to identify that the condition was significantly more severe in CLP mice compared with control mice. The survival rate and severity score of CLP mice were deteriorated after the CLP challenge (Figures 1D and 1E; 30.00% septic mice, 100.00% sham mice survived). We continuously recorded electrocardiograms for 10 days after CLP challenge and found that the total number of arrhythmias and the incidence of ventricular arrhythmias (VA) reached the peak at 12 h (50%, 50%, respectively; Figures 1F, 1H, and 1I) but did not find atrial fibrillation (AF). Increased fibrosis was found in CLP mice heart (Figure 1G). Meanwhile, heart rate decreased, and PR intervals, QRS intervals, and QTc intervals were all significantly longer in septic mice than sham mice, which indicated a prolonged conduction from atrial to ventricle, ventricular depolarization and repolarization (Figure 1I). In addition, biochemistry analyses of serum demonstrated significantly enhanced levels of lactate dehydrogenase (LDH), creatine kinase-MB (CK-MB), and cardiac troponin I (cTnI) in CLP mice when compared with control mice (Figure 1J). More importantly, an increased collagen volume was found in the CLP group. Western blot found reduced Nav1.5, Cx40 levels and increased Cav1.2, Kv4.2, Serca-2A, Cx43, IL-10, IL-1β, tumor necrosis factor alpha (TNF-α) in 12 h after CLP survey challenge (Figure 1K). Next, we performed DNA extraction and PCR of content from colon, ileum, and cecum of CLP mice with or without arrhythmia to explore the potential association between *C. sakazakii* (Cs) and the occurrence and development of sepsis arrhythmia. Specifically, an increased abundance of *C. sakazakii* (Cs) was observed in the colon, ileum, and cecum content of the CLP mice with arrhythmia compared with the no arrhythmia mice, especially in the cecum (Figure 1M). These findings indicated that *C. sakazakii* (Cs) was increased in CLP mice with arrhythmia and positively correlates with arrhythmia caused by sepsis. In agreement with these findings, it has been reported that *Cronobacter* or its phage can predict ventricular arrhythmias in patients with sepsis-induced cardiomyopathy.^8^

### *C. sakazakii* and its culture supernatant directly triggered arrhythmia

**Figure 2:**
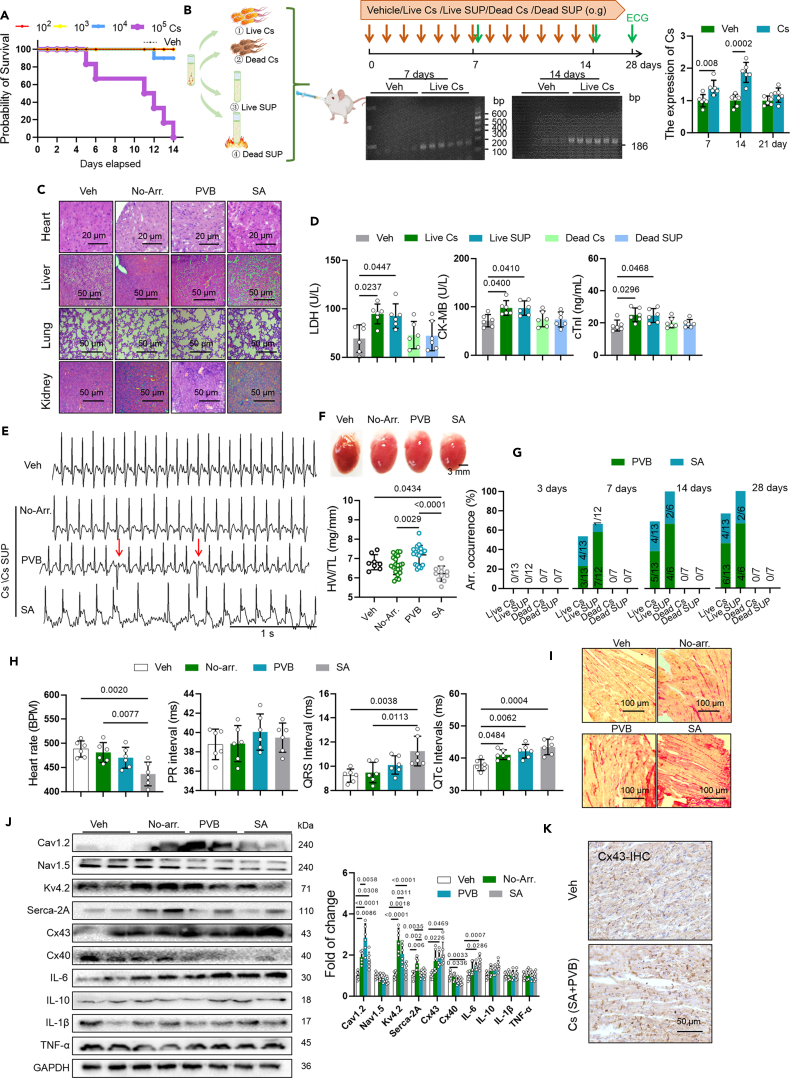
*C*. *sakazakii* and its culture supernatant could directly trigger arrhythmia (A) Probability of survival was calculated for mice treated with different dose of *C. sakazakii*. (B) Experimental design for *C. sakazakii* treatment and surface ECG testing. And relative levels of *C*. *sakazakii* extracted from feces of live *C*. *sakazakii*-treated mice in 7, 14, and 12 days were evaluated by PCR (*n* = 6/per group). Data are presented as the mean ± SD. Data analyzed by unpaired two-tailed Student’s t test. (C) Representative H&E staining images of kidney, lung, liver, and heart in each group. (D) Cardiac LDH, CK-MB, and cTnI levels were quantified using commercial assay kits (*n* = 6/per group). Data are presented as the mean ± SD. Data analyzed by one-way ANOVA with Tukey’s post-hoc test. (E) Sample surface ECG traces of two different types of arrhythmic events: premature ventricular beats (PVB), sinus arrhythmia (SA). Scale bar: 1 s. (F) The overall heart size. Scale bar: mm. And quantitative analysis of heart weight/tibia length (HW/TL) in the different groups. Data are presented as the mean ± SD. Data analyzed by one-way ANOVA with Tukey’s post-hoc test. (G) Summary the occurrence of PVB and SA in mice treated by PBS, live *C. sakazakii* (live Cs), autoclaving-killed *C. sakazakii* (dead CS), live *C. sakazakii* supernatant (live SUP), and autoclaving-killed *C. sakazakii* supernatant (dead SUP). And numbers in parentheses indicate the number of mice that occurred SA and PVB in 7 and 14 days. (H) Differences in heart rates, PR intervals, QRS intervals, and QTc intervals among each group. Data are presented as the mean ± SD. Data analyzed by one-way ANOVA with Tukey’s post-hoc test. (I) Ventricular fibrosis in mice that occurred SA and PVB was measured by Sirius red staining. (J) Representative western blot and quantification of Cx43, Cx40, Nav1.5, Cav1.2, Serca-2A, Kv4.2, IL-6, IL-10, IL-1β, TNF-α, and GAPDH in ventricle of vehicle-treated, no arrhythmia, PVB, and SA mice. And, GAPDH was used for internal normalization. Data are presented as the mean ± SD. Data analyzed by one-way ANOVA with Tukey’s post-hoc test. (K) Immunohistochemistry image of Cx43 was used to detect myocardial injury and gap junction remodeling in ventricle of vehicle-treated, PVB, and SA mice. Veh, vehicle; IHC, immunohistochemistry.

To investigate whether *C. sakazakii* (Cs) directly take part in arrhythmogenesis, we treated 8-week-old CD-1 mice with 10^2^, 10^3^, 10^4^, and 10^5^ *C. sakazakii* (Cs) for two weeks. Only 10^2^ and 10^3^ *C. sakazakii* (Cs) treatment were not lethal (Figure 2A), so we chose10^2^ for subsequent experiments to demonstrate the potential association between *C. sakazakii* and arrhythmia. We treated 8-week-old CD-1 mice with PBS, live *C. sakazakii* (live Cs), autoclaving-killed *C. sakazakii* (dead CS), live *C. sakazakii* supernatant (live SUP), autoclaving-killed *C. sakazakii* supernatant (dead SUP) for 14 days, and then no treatment for 7 days (Figure 2B). We then evaluated the impact of *C. sakazakii* (Cs) and supernatant on sepsis-associated damage and arrhythmia. Enhanced *C. sakazakii* (Cs) DNA levels in fecal samples were observed in mice treated with live *C. sakazakii* (live Cs) on days 7 and 14, but no significant changes were detected on day 21, compared with those from mice treated with PBS (Figure 2B). Histomorphological changes are found using H&E staining, which showed normal liver, lung, kidney, and heart structure in PBS-treated mice but disorder of myocardial fibers accompanied by irregular mass in mice treated with live *C. sakazakii* (live Cs) and live *C. sakazakii* (Cs SUP) supernatant (Figure 2C). In addition, biochemistry analyses of serum showed obviously elevated levels of LDH, CK-MB, and cTnI in live mice treated with *C. sakazakii* (live Cs) and live *C. sakazakii* (Cs SUP) supernatant, when compared with control mice (Figure 2D). There was no arrhythmia after 3 days’ treatment of *C. sakazakii, C. sakazakii supernatant*, dead *C. sakazakii*, or dead *C. sakazakii* supernatant treatment until workend. We captured spontaneous SA, PVB in live *C. sakazakii*, live *C. sakazakii* supernatant-treated mice in 7 and 14 days (Figure 2D). We found that the heart weight to body weight ratio (HW/BW) increased in PVB mice, but decreased in SA mice, whereas none of the control animals displayed spontaneous arrhythmia (Figures 2E and 2F). When stopped the treatment of live *C. sakazakii* and live *C. sakazakii* supernatant, we still captured spontaneous SA, PVB in live *C. sakazakii*, live *C. sakazakii* supernatant-treated mice in 21 days (Figure 2G), which indicated that *C. sakazakii* trigger arrhythmia by irreversible myocardial damage. Meanwhile, heart rate was decreased, and QRS intervals and QTc intervals were both significantly prolonged in mice with arrhythmia, which indicated a prolonged conduction from atrial to ventricle, ventricular depolarization and repolarization (Figure 2H). An increased myocardial fibrosis and Cx43 remodeling (remained at side-to-side junctions) were found in mice with arrhythmia (Figures 2I and 2K). More importantly, western blot analysis found that the levels of connexin40 (Cx40) reduced and Cav1.2, Kv4.2, connexin43 (Cx43), and interleukin-6 (IL-6) increased in mice treated by live *C. sakazakii* and its supernatant (Figure 2J). Next, we confirmed it by measuring the expression and distribution of Cx43 in mouse ventricle using immunohistochemically staining (Figure 2I). Importantly, in agreement with western blotting, as presented in Figure 2J, *C. sakazakii* enhanced Cx43 protein, decreased localization at the ventricular intercalated disc with scattered structures, swelled plaques, and little remodeling. These results indicate that *C. sakazakii* (Cs) is involved in arrhythmogenesis through a certain component in its supernatant. Thus, we hypothesized that outer membrane vesicles of *C. sakazakii* mediated arrhythmogenesis.

Based on the above results, we administered gap junction blocker carbenoxolone (CBX) in 8-week-old CD-1 mice for 3 days and then oral gavaged with *C. sakazakii*. As expected, CBX reduced the occurrence of arrhythmia induced by Cs (Figure S1A). Meanwhile, we constructed the CLP model after CBX pretreatment. In addition, biochemistry analyses of serum demonstrated significantly reduced ALT and AST levels in CBX-treated CLP mice when compared with CLP mice (Figure S1B). CBX significantly reduced the mortality and arrhythmia caused by CLP (Figures S1C and S1F). Furthermore, severity score was also found to be lower in CBX-pretreated mice compared with sham mice after the CLP challenge (Figure S1C). More importantly, *C. sakazakii* (Cs) and supernatant-induced arrhythmia were reduced or restored when pretreated with CBX. Serum biochemistry analyses showed reduced LDH, CK-MB, and cTnI levels in CBX-treated CLP mice than CLP mice (Figure S1F). These results indicated that gap junction regulation could improve arrhythmia caused by *C. sakazakii* and sepsis, which was consistent with recent study that decreased Panx1 by CBX inhibited inflammatory cytokines and apoptosis via inhibiting NLRP3 inflammasome activation and regulating apoptotic protein Bax and Bcl_2_ levels, respectively.^16^

### Outer membrane vesicles of *C. sakazakii* triggered arrhythmia in mice

**Figure 3:**
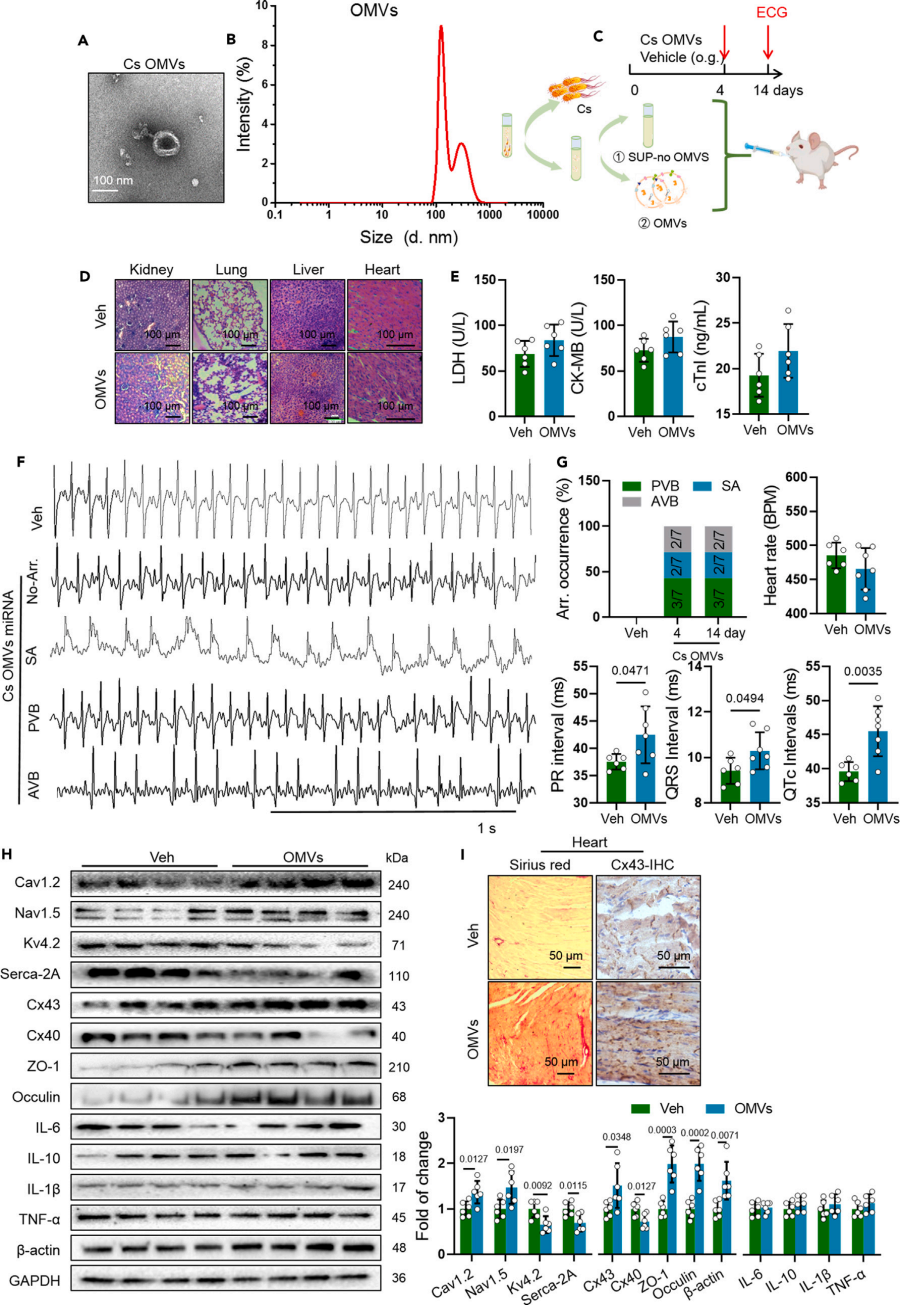
Outer membrane vesicles of *C. sakazakii*-triggered arrhythmia in mice (A) Transmission electron microscopy (TEM) image of purified *C. sakazakii*-derived OMVs (outer membrane vesicles). Scale bar = 100 nm. (B) Size distribution of OMVs according to diameter. (C) Experimental design for *C. sakazakii*-derived OMVs treatment and surface ECG testing. (D) Representative H&E staining images of kidney, lung, liver, and heart in mice treated with PBS and outer membrane vesicles from *C. sakazakii* (OMVs). (E) Cardiac LDH, CK-MB, and cTnI levels were quantified using commercial assay kits. Data are presented as the mean ± SD. Data analyzed by unpaired two-tailed Student’s t test. (F) Sample surface ECG traces of three different types of arrhythmic events: premature ventricular beats (PVB), sinus arrhythmia (SA), and atrioventricular block (AVB). Scale bar: 1 s. (G) Summary the occurrence of SA, PVB, AVB, heart rates, PR intervals, QRS intervals, and QTc intervals in mice treated with PBS and outer membrane vesicles derived from *C. sakazakii* (OMVs). Numbers in parentheses indicate the number of mice that occurred SA, PVB, and AVB following OMV treatment. Data are presented as the mean ± SD. Data analyzed by unpaired two-tailed Student’s t test. (H) Representative western blot and quantification of Cx43, Cx40, Nav1.5, Cav1.2, Serca-2A, Kv4.2, IL-6, ZO-1, occludin, IL-10, IL-1β, TNF-α, and GAPDH in ventricle of vehicle and *C. sakazakii*-derived OMV-treated mice. GAPDH was used for internal normalization. Data are presented as the mean ± SD. Data analyzed by unpaired two-tailed Student’s t test. (I) Immunohistochemistry (IHC) image of Cx43 and Sirius red staining was used to detect gap junction remodeling and fibrosis in the left ventricle of vehicle and *C. sakazakii*-derived OMV-treated mice.

The bacterial outer membrane vesicles (OMVs) serve as one of the mechanisms that commensal bacteria utilize to modulate host responses and diseases.^17^ We purified *C. sakazakii*-derived OMVs from culture supernatant, which was free from bacterial cell debris and flagella, and then were identified as round particles with a typical cup-shaped morphology (Figure 3A). The size of OMVs was approximately 100 nm in diameter (Figure 3B), which was consistent with the previous report.^18^ To test the above hypothesis, 8-week-old CD-1 male mice were oral gavaged (o.g.) with PBS, OMVs (50 μg), followed by analysis of ECG at 4 and 14 days, as shown in Figure 3C. Four days after OMV oral administration (0.5 mg/20g) in mice, disordered myofilament arrangement was found by H&E staining (Figure 3D). In addition, no difference was found in LDH, CK-MB, and cTnI levels in OMVs-treated mice than control mice (Figure 3E). Moreover, spontaneous SA (42.85% and 42.85%, respectively), PVB (28.57% and 28.57%, respectively), and AVB (28.57% and 28.57%, respectively) were captured in *C. sakazakii*-derived OMV-treated mice in 4 and 14 days (Figures 3F and 3G). Meanwhile, the PR intervals, QRS intervals, and QTc intervals were all significantly longer in *C. sakazakii*-derived OMV-treated mice than PBS-treated mice, which indicated a prolonged conduction from atrial to ventricle, ventricular depolarization and repolarization (Figure 3G). More importantly, western blot result showed *C. sakazakii*-derived OMVs increased Cav1.2, Nav1.5, Cx43, ZO-1, occludin, and β-actin level and decreased Kv4.2 and Serca-2A level but did not find any change in inflammatory cytokines (Figure 3H). Next, the expression and distribution of Cx43 in ventricle was measured by immunohistochemistry staining. We found that OMVs enhanced Cx43 level and led to gap junction remodeling and cardiac fibrosis (Figure 3I). In conclusion, these results demonstrated that *C. sakazakii*-derived OMVs were involved in myocardial injury and arrhythmogenesis.

**Figure 4:**
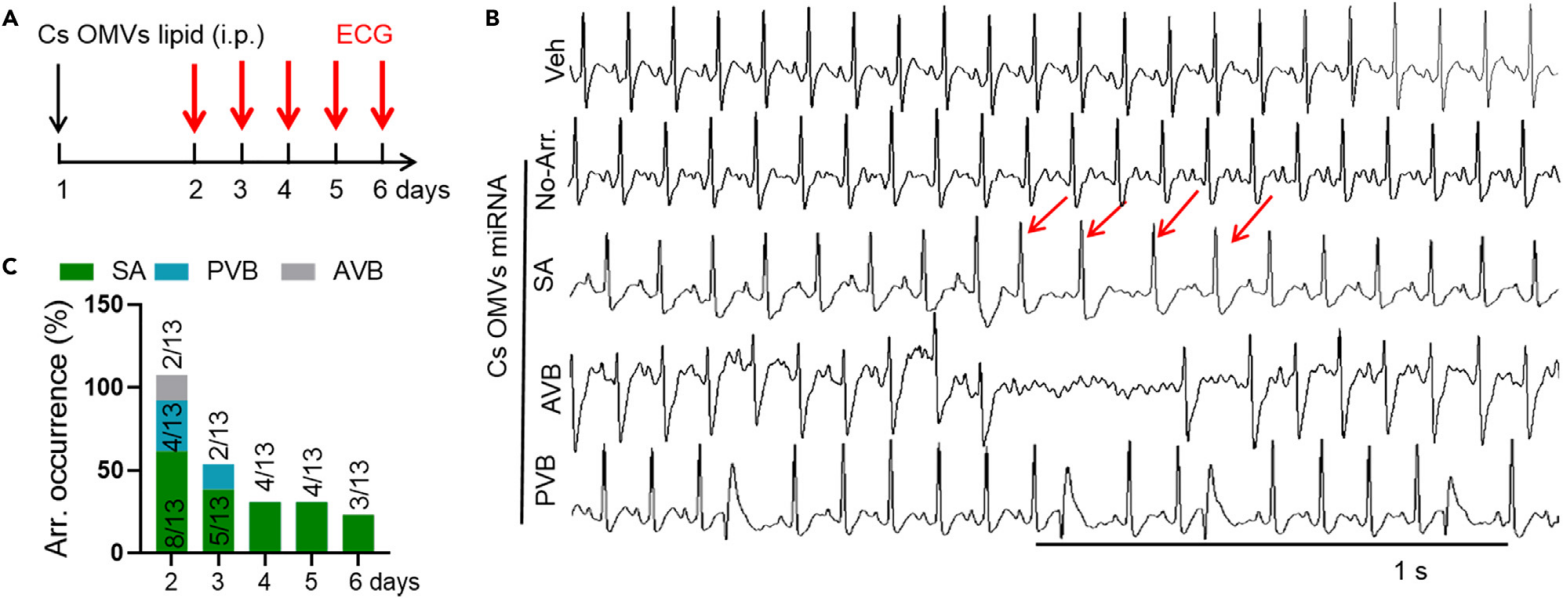
The lipid from *C. sakazakii*-promoted arrhythmogenesis (A) Experimental design for *C. sakazakii*-derived OMV lipid treatment and surface ECG testing. (B) Sample surface ECG traces of three different types of arrhythmic events: premature ventricular beats (PVB), sinus arrhythmia (SA), and atrioventricular block (AVB). Scale bar: 1 s. (C) Summary of the occurrence of SA, PVB, and AVB in mice treated with lipid from *C. sakazakii*-derived OMVs. Numbers in parentheses indicate the number of mice that occurred SA, PVB, AVB following lipid treated.

Meanwhile, to explore whether OMVs-lipid is involved in arrhythmogenesis, mice were oral gavaged with lipid extracted from OMVs (Figure 4A). We captured SA and PVB after OMVs-lipid treatment. The incidence of arrhythmia decrased with the time going (Figures 4B and 4C), indicating that lipid from *C. sakazakii*-derived OMVs can trigger arrhythmia directly.

**Figure 5:**
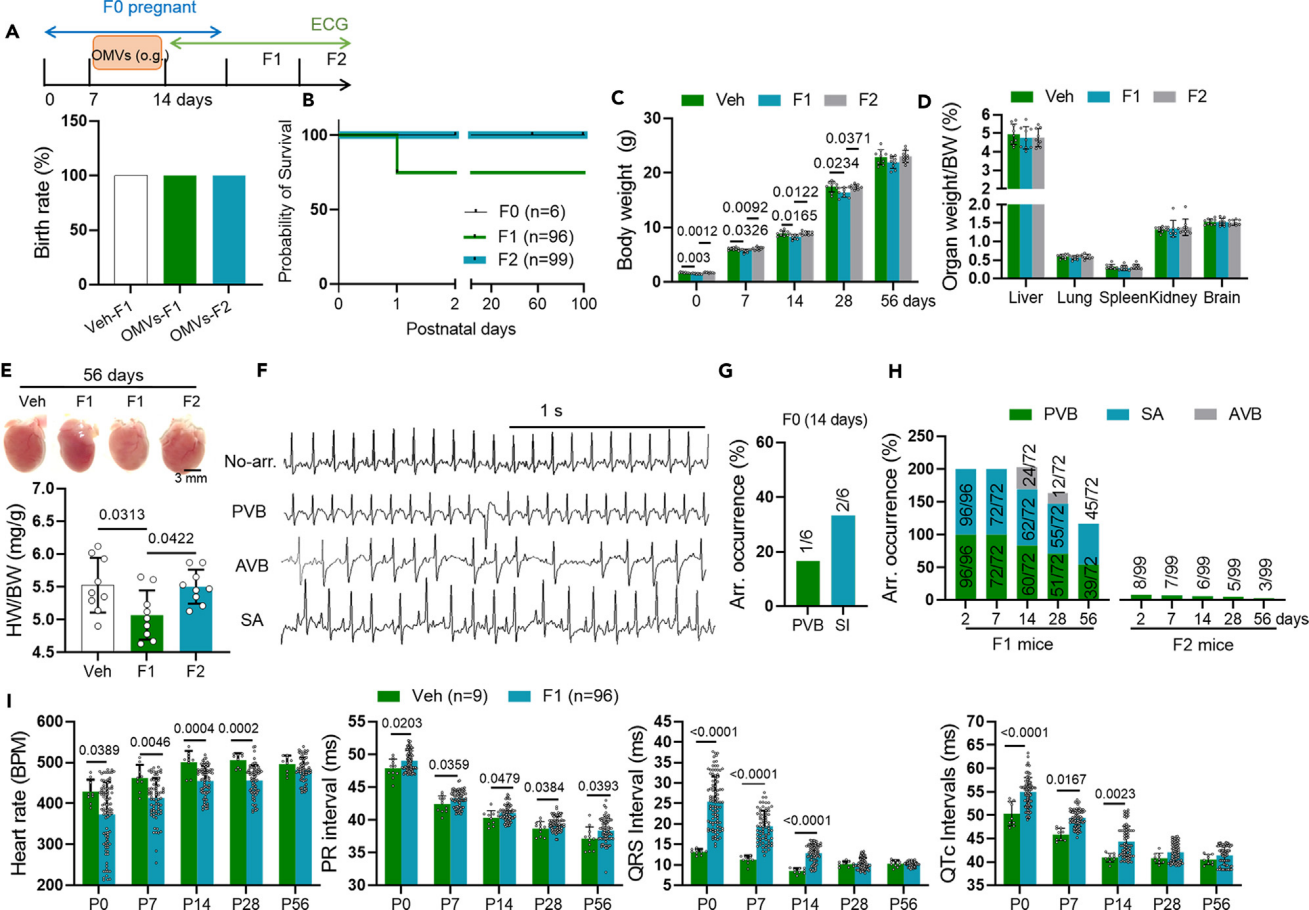
*C. sakazakii*-derived OMVs treatment in pregnant mice promoted arrhythmia in offspring (A) Experimental design for *C. sakazakii*-derived OMVs treatment in pregnant mice model and surface ECG testing. (B) Analysis of the mouse birth rate. (C–E) The overall heart size. Scale bar: 3 mm. Quantitative analysis of body weight, organ (liver, lung, spleen, kidney, and brain) weight/body weight, and heart/body weight (HW/BW) in the different groups. Data are presented as the mean ± SD. Data analyzed by one-way ANOVA with Tukey’s post-hoc test. (F) Sample surface ECG traces of three different types of arrhythmic events: premature ventricular beats (PVB), sinus arrhythmia (SA), and atrioventricular block (AVB). Scale bar: 1 s. (G and H) The summary of total arrhythmias, PVB, and VT in each group. Data analyzed by unpaired two tailed Student’s t test. Data analyzed by unpaired two-tailed Student’s t test. (I) Differences in heart rates, PR intervals, QRS intervals, and QTc intervals were showed among each group. Data are presented as the mean ± SD. Data analyzed by unpaired two-tailed Student’s t test.

To further verify the long-term role of *C. sakazakii*-derived OMVs in cardiac electrophysiology, we administered OMVs (0.1 mg/20g body weight) to early pregnant mice. As shown in Figures 5A and 5B, neonatal death, instead of embryonic death, was significantly elevated in F1 mice. Twenty-five percent of F1 mice suffered sudden cardiac death within one day after birth, whose mother was treated with OMVs (Figure 5B). Compared to vehicle mice, F1 mice, whose mother was treated by OMVs, exhibited a significant reduction in both body weight and HW/BW. Whereas, no alterations of liver weight, lung weight, spleen weight, kidney weight, and brain weight to body weight were observed among different groups (Figures 5C–5E). In addition, spontaneous SA, PVB was captured in OMV-treated F0 mice in 14 days, and serious SA, PVB and AVB was captured in F1 mice but only small occurrence of PVB in F2 mice (Figures 5F–5H). Meanwhile, heart rate decreased, and PR intervals, QRS intervals, and QTc intervals were all significantly longer in F1 mice, whose mother was treated by OMVs, than vehicle mice, which indicated a prolonged conduction from atrial to ventricle, ventricular depolarization and repolarization (Figure 5I). Taken together, we demonstrated that *C. sakazakii*-derived OMVs could trigger arrhythmias across the placental barrier.

### *C*. *sakazakii* and its OMVs increased arrhythmia caused by sepsis

**Figure 6:**
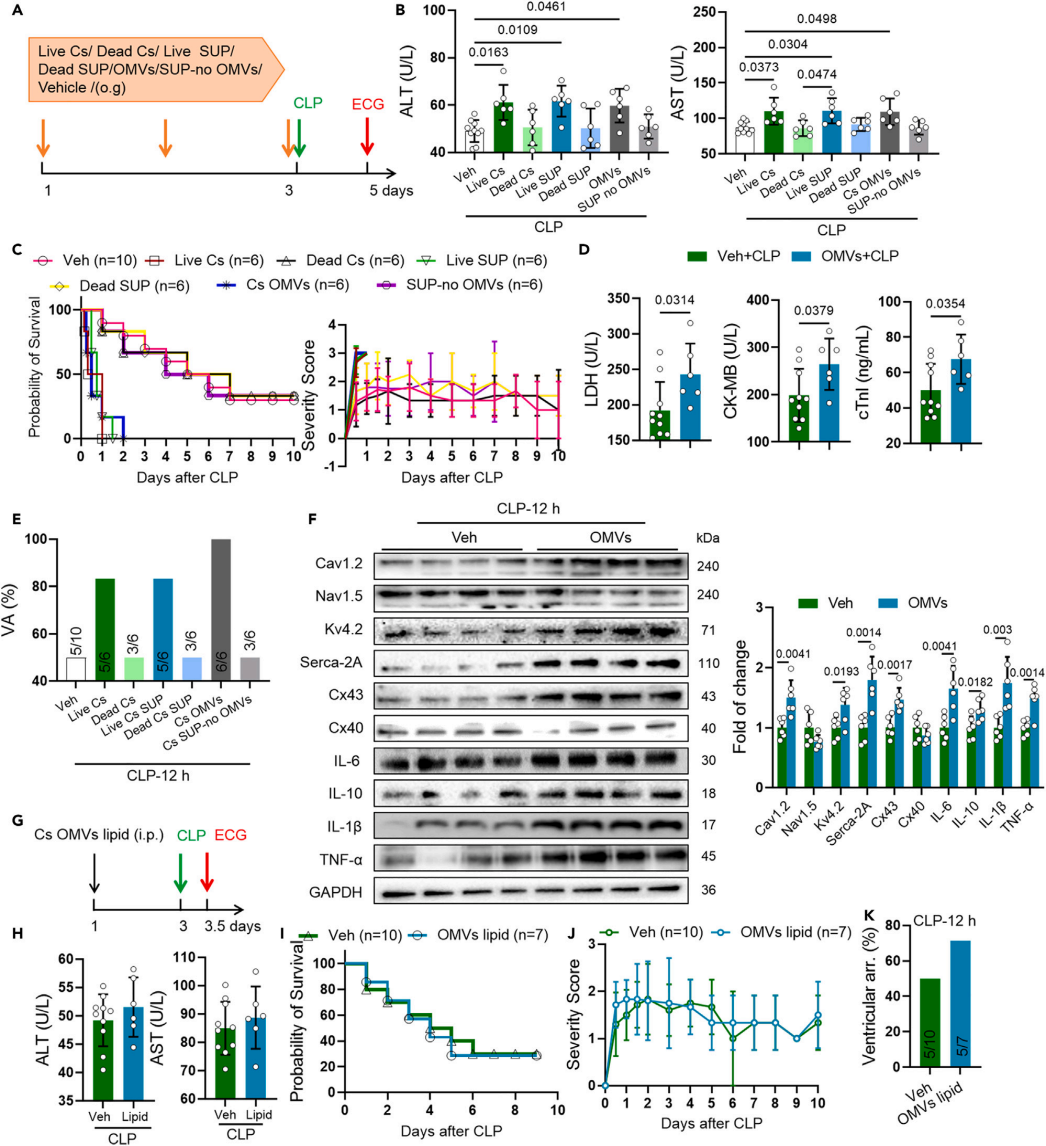
*C*. *sakazakii* and its OMVs increased arrhythmia caused by sepsis (A) Experimental design for CLP mice and treatment of PBS, live *C. sakazakii* (live Cs), autoclaving-killed *C. sakazakii* (dead CS), live *C. sakazakii* supernatant (live SUP), autoclaving-killed *C. sakazakii* supernatant (dead SUP), and surface ECG testing. (B and H) Serum AST and ALT levels were quantified using commercial assay kits. Data are presented as the mean ± SD. Data analyzed by one-way ANOVA with Tukey’s post-hoc test in (B) and by unpaired two-tailed Student’s t test in (H). (C, I, and J) Probability of survival and severity score was calculated for each group. (D) Cardiac LDH, CK-MB, and cTnI levels were quantified using commercial assay kits. Data are presented as the mean ± SD. Data analyzed by unpaired two-tailed Student’s t test. (E and K) Summary of the occurrence of ventricular arrhythmia (VA) was showed in different group. (F) Representative western blot and quantification of Cx43, Cx40, Nav1.5, Cav1.2, Serca-2A, Kv4.2, IL-6, IL -10, IL -1β, TNF-α, and GAPDH in ventricle of vehicle and *C. sakazakii*-derived OMV-treated CLP mice (*n* = 6). GAPDH was used for internal normalization. Data are presented as the mean ± SD. Data analyzed by unpaired two-tailed Student’s t test. (G) Experimental design for *liqid of C. sakazakii* OMVs treatment in CLP mice and surface ECG testing.

To further clarify the involvement of *C. sakazakii* (Cs) and its OMVs in sepsis arrhythmia and progression, we treated 8-week-old CD-1 male mice with PBS, live *C. sakazakii* (live Cs), autoclaving-killed *C. sakazakii* (Cs) (dead Cs), live *C. sakazakii* (Cs) supernatant (live SUP), autoclaving-killed *C. sakazakii* (Cs) supernatant (dead SUP), *C. sakazakii*-derived OMVs, or *C. sakazakii* (Cs) supernatant without OMVs (SUP-no OMVs) followed by sham or CLP surgery (Figure 6A). Then, we evaluated the impact of *C. sakazakii* (Cs), supernatant, and OMVs on sepsis-associated damage and arrhythmia. More importantly, CLP-induced injury was significantly aggravated in live *C. sakazakii* (Cs), live *C. sakazakii* (Cs) supernatant, and *C. sakazakii*-derived-OMV-treated mice, as evidenced by increased mortality rate and hepatic inflammatory factors (ALT and AST), survival rate, and severity score (Figures 6B and 6C). Accordingly, CLP-induced elevation levels of myocardial injury factor (LDH, CK-MB, cTnI) and ventricular arrhythmia were further compromised in mice with live *C. sakazakii* (live Cs), live *C. sakazakii* supernatant (live SUP), and *C. sakazakii*-derived OMVs (Figures 6D and 6E). Meanwhile, live *C. sakazakii* (Cs), live *C. sakazakii* (Cs) supernatant, and *C. sakazakii*-derived OMV treatment also exacerbated abnormal ion channel protein expression in CLP mice (Figure 6F). Taken together, we demonstrate that live *C. sakazakii* (Cs), live *C. sakazakii* (Cs) supernatant, and *C. sakazakii*-derived OMVs, but not dead *C. sakazakii* (Cs), dead supernatant, or *C. sakazakii* (Cs) supernatant without OMVs, could deteriorate arrhythmia, the cardiac damage, and lethality caused by sepsis.

Meanwhile, to explore whether OMVs-lipid is involved in arrhythmogenesis, mice were intraperitoneally injected with lipid extracted from OMVs, prior to CLP surgery (Figure 6L). In addition, biochemistry analyses of serum demonstrated no significant changes of ALT and AST levels were found between groups (Figure 6M). More importantly, the overall incidence of ventricular arrhythmia (VA) was markedly increased (70.0% in OMVs vs.50.0% in vehicle) but no changes in severity score in lipid-treated mice after the CLP challenge (Figures 6N and 6O). Taken together, we demonstrate that lipid from *C. sakazakii*-derived OMVs could deteriorate arrhythmia caused by sepsis.

### Short-chain fatty acids decreased the occurrence of arrhythmia caused by *C. sakazakii* and sepsis

**Figure 7:**
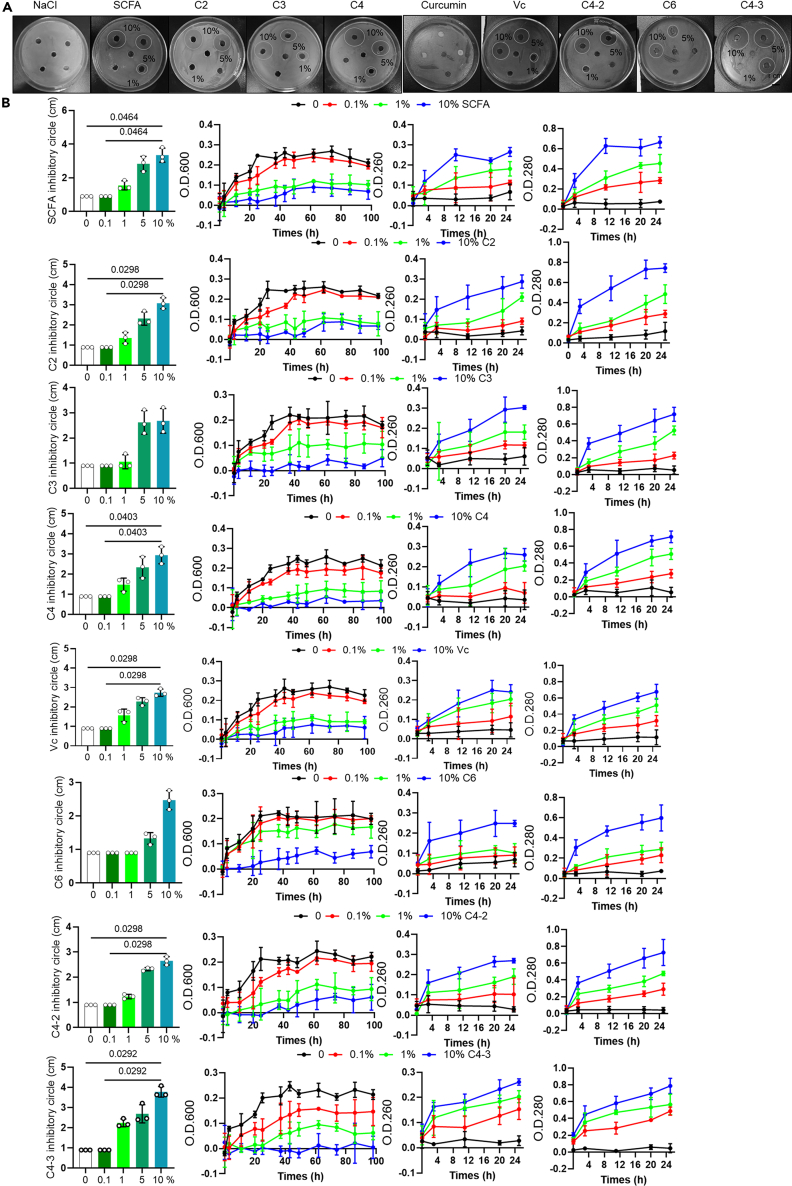
The antibacterial activity of short-chain fatty acids (A) Inhibition zones around short-chain fatty acids (SCFA), sodium acetate (C2), sodium propionate (C3), sodium butyrate (C4), succinate (C4-2), tartaric acid (C4-3), adipic acid (C6), and vitamin C (Vc) against *C. sakazakii* (the middle hole is the negative sample). (B) Inhibition zones diameter, bacterial growth inhibition curve, and leakage of cytoplasm and nucleic acid for *C. sakazakii* up to 92 h with different concentrations (10%, 5%, 1%,0.1%) of SCFA, sodium acetate (C2), sodium propionate (C3), sodium butyrate (C4), succinate (C4-2), tartaric acid (C4-3), adipic acid (C6), vitamin C (Vc), and medium-only (negative control). Data are presented as the mean ± SD. Data analyzed by one-way ANOVA with Tukey’s post-hoc test in bacterial growth inhibition curve.

To explore the therapeutic drugs for *C. sakazakii* (Cs)-induced arrhythmia, we screened *C. sakazakii* antibacterial drugs. The effect of SCFAs and vitamin C on *C. sakazakii* (Cs) viability was studied *in vitro* by varying time of incubation and concentration. To avoid the effect of osmotic pressure on bacteria, sodium chloride solution was used as a control. The results of inhibition zone diameter, extracellular ultraviolet absorption, and leakage of cytoplasm and nucleic acid showed that every SCFAs obviously reduced *C. sakazakii* survival rate (Figures 7A and 7B), indicating that multiple SCFAs have good antimicrobial activities against *C. sakazakii* *ex vivo*.

**Figure 8:**
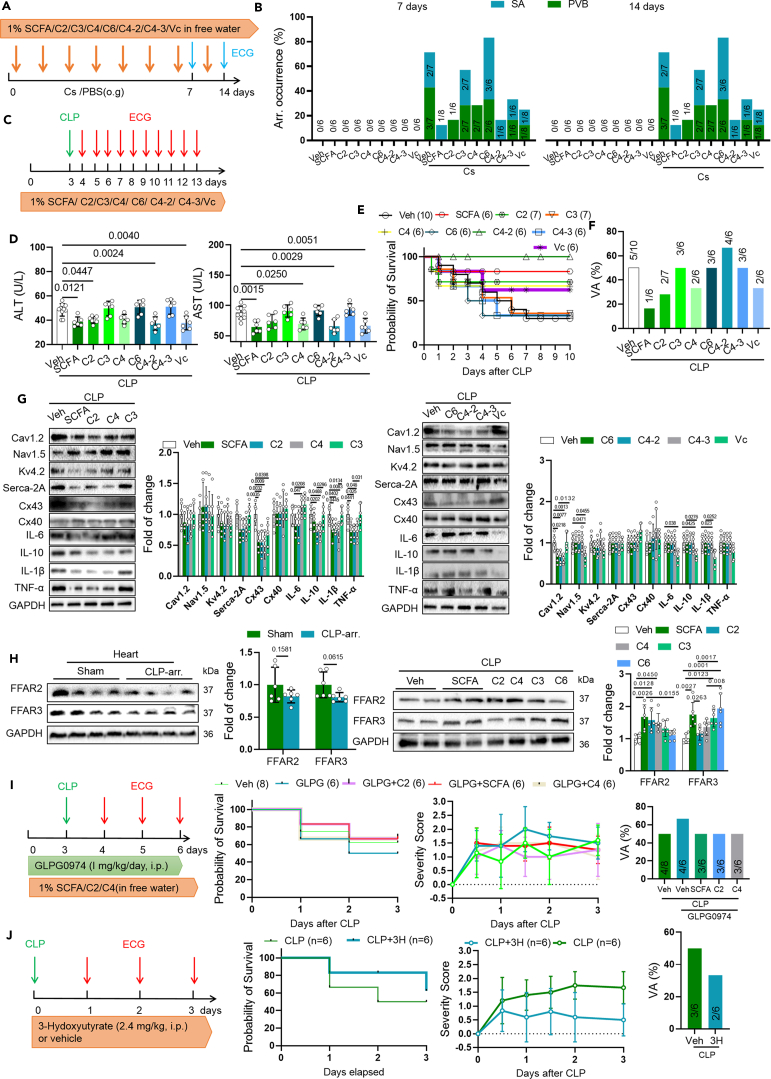
Short-chain fatty acids decreased the occurrence of arrhythmia caused by *C. sakazakii* and sepsis (A) Experimental design for SCFA, sodium acetate (C2), sodium propionate (C3), sodium butyrate (C4), succinate (C4-2), tartaric acid (C4-3), adipic acid (C6), vitamin C treatment and *C. sakazakii* oral gavage, and surface ECG testing. (B) Summary of the occurrence of SA, PVB in septic mice treated with SCFA, sodium acetate (C2), sodium propionate (C3), sodium butyrate (C4), succinate (C4-2), tartaric acid (C4-3), adipic acid (C6), and vitamin C (Vc). Numbers in parentheses indicate the number of mice that occurred SA, PVB. (C) Experimental design for SCFA, sodium acetate (C2), sodium propionate (C3), sodium butyrate (C4), succinate (C4-2), tartaric acid (C4-3), adipic acid (C6), vitamin C (Vc) treatment in CLP mice, and surface ECG testing. (D) Serum AST, ALT levels were quantified using commercial assay kits. Data are presented as the mean ± SD. Data analyzed by one-way ANOVA with Tukey’s post-hoc test. (E–G) Probability of survival and sepsis severity score were calculated for each group. (G) Representative western blot and quantification of Cav1.2, Nav1.5, Kv4.2, Serca-2A, Cx43, Cx40, IL-6, IL-10, IL-1β, TNF-α, FFAR2, FFAR3, and GAPDH in ventricle of SCFA, sodium acetate (C2), sodium propionate (C3), sodium butyrate (C4), succinate (C4-2), tartaric acid (C4-3), adipic acid (C6), vitamin C (Vc) treated CLP mice (*n* = 6). GAPDH was used for internal normalization. Data are presented as the mean ± SD. Data analyzed by one-way ANOVA with Tukey’s post-hoc test. (H) Experimental design for SCFA, sodium acetate (C2), sodium butyrate (C4), succinate (C4-2), and GLPG 0794 treatment in CLP mice, and surface ECG testing. Probability of survival, severity score, and occurrence of ventricular arrhythmia were calculated for each group. (I and J) Experimental design for GLPG0974 and 3-hydoxybutyrate treatment in CLP mice and surface ECG testing. Probability of survival, severity score, and occurrence of ventricular arrhythmia were calculated for each group. Data are presented as the mean ± SD.

To explore the potential therapeutic effect of SCFAs, C2, C3, C4, C4-2, C4-3, C6, and vitamin C in arrhythmia caused by *C. sakazakii* (Cs), we administered them to mice before *C. sakazakii* (Cs) oral gavage (Figure 8A). Though C6 aggravated arrhythmia, and C3 mildly reduced arrhythmia, C6 aggravated arrhythmia, and C3 reduce arrhythmia, SCFAs reduced the occurrence of arrhythmia (Figure 8B), indicating that SCFAs could decrease occurrence of arrhythmia by reducing *C. sakazakii*.

To investigate the therapeutic effect of SCFAs, C2, C3, C4, C4-2, C4-3, C6, and vitamin C in sepsis-associated arrhythmia, we administered them to mice for 3 days prior to CLP surgery (Figure 8C). Serum biochemistry analyses showed reduced ALT and AST levels in SCFA-, C2-, C4-, C4-2-, and vitamin C-treated CLP mice than CLP mice (Figure 8D). In addition, survival analysis revealed that pretreatment with SCFAs, C2, and C4 prolonged the survival of septic mice compared to those pretreated with the vehicle (Figure 8E). Only SCFAs, C2 and C4, and vitamin C reduced the occurrence of VA (Figure 8F). In agreement with these findings, in order to assess whether the antibacterial properties of SCFAs are associated with ion channel protein, we tested the effects of SCFAs on the level of various ion channel protein in heart (Figure 8G). More importantly, pretreatment with SCFAs, C2, and C4 significantly alleviated severity score and death rate, restored Cx43 expression, and decreased inflammatory cytokines expression in the heart from CLP septic mice (Figure 8G). Collectively, our results indicate that SCFAs could protect against arrhythmia and lethal in septic mice.

SCFAs exert their effects mainly via two GPCR types, FFAR2 and FFAR3. More specifically, SCFAs, particularly C2 and C4 that reduced arrhythmogenesis beyond their anti-microbial effect, are known to activate FFAR2 and FFAR3, two G-protein-coupled receptors with documented cardiovascular effects.^19^ To investigate the involvement of these receptors in the protective effects of SCFAs against sepsis-associated arrhythmia, we measured the levels of FFAR2 and FFAR3 using western blot. Firstly, we found a non-significant mild reduced expression of FFAR2 and FFAR3 after a 12-h CLP challenge compared to sham operation mice. Then, we found pretreatment by SCFAs, C2, C3, and C4 could increase the expression of FFAR2, whereas C3, C4, and C6 increased FFAR3 expression (Figure 8H), which is consistent with recent reports indicating that C2 prefers FFAR2 while C6 prefers FFAR3.^19^ To investigate the role of FFAR2 in the anti-arrhythmic effects of C2 and C4, we pretreated mice with the FFAR2 inhibitor (GLPG0974) for consecutive three days, along with SCFAs, C2, or C4, before performing CLP surgery. We found that blocking FFAR2 by GLPG0974 abolished the anti-arrhythmic and sepsis-protective effects of SCFAs, C2, and C4 (Figure 8I), indicating that the anti-arrhythmic effect of SCFAs in sepsis is partly dependent on FFAR2.

Small ketone bodies, such as 3-hydroxybutyrate, have been postulated to exert cardioprotective effects, including against arrhythmias.^20^ We aimed to investigate whether 3-hydroxybutyrate could reduce sepsis damage and arrhythmias, thus we administered 3-hydroxybutyrate at a dose of 2.4 mg/kg (i.p.) once a day to mice after CLP surgery. We found that 3-hydroxybutyrate treatment reduced sepsis mortality, improved sepsis severity score, and decreased the incidence of post-sepsis arrhythmias. These results suggest that 3-hydroxybutyrate has a certain therapeutic effect on arrhythmias caused by sepsis.

## Discussion

The gut microbiota is considered to be one of the upstream regulators of sepsis; however, the exact role of intestinal flora in sepsis remains unclear.^8^ Further studies should be conducted on functional microbial patterns to elucidate the pathogenesis septic cardiomyopathy. Here, we found an association between *C. sakazakii*-derived OMVs and sepsis-induced arrhythmia in a mouse sepsis model. In addition, we found that OMVs can also induce arrhythmias across the placental barrier. And its arrhythmia mechanism mainly involves the deregulation of myocardial ion channel protein expression. For example, OMVs increase the sodium channel protein Nav1.5 and calcium channel protein Cav1.2, decrease the potassium channel protein Kv4.2, and increase Cx43, ZO-1 and occludin level, but decrease the expression of Cx40. Finally, we observed sodium acetate and sodium butyrate were able to diminish arrhythmia caused by *C. sakazakii* and sepsis.

Sepsis enhances the peak value of the Na current, decreases the late Na current, increases the peak depolarization velocity, prolongs the repolarization time, and ultimately leads to a decrease in cardiomyocyte excitability and shortened action potential duration (APD).^21^ Activation of K-ATP channels resulted in an enhanced delayed rectifier K^+^ current and a reduced small-conductance calcium-activated potassium current and enhanced Ito currents also closely associated with the occurrence of supraventricular tachyarrhythmia.^22^ The impairment of myocardial contractile function is also attributed to the reduction in calcium transient amplitude, the peak of L-type calcium current, and the enhancement of Na/Ca-exchanger currents.^23^^,^^24^ The changes in ion channels lead to the occurrence of various types of arrhythmias. In our study, it was also confirmed that aberrant ion channel proteins expression involved in the occurrence sepsis arrhythmia. And, one of the mechanisms of arrhythmia induced by *C. sakazakii*-derived OMVs and live *C. sakazakii* was dysregulation of cardiac ion channel proteins expression.

We found a positive correlation between *C. sakazakii* and ventricular arrhythmias in septic mice. However, with further study, oral administration of *C. sakazakii* led to not only ventricular arrhythmias but also sinus arrhythmia and even sick sinus syndrome. This may be due to the complex regulation of sympathetic nervous system in septic cardiomyopathy. Sepsis is characterized by hyperactivation of sympathetic nervous system and increased levels of circulating endogenous catecholamine and increased stimulation of adrenergic receptor, resulting in desensitization, which may cover up the sinus irregularity induced by *C. sakazakii*. These processes are further enhanced by the effect of inflammatory mediators and bacterial toxins.

We feel confused that *C. sakazakii* or its supernatant treatment only triggered PVB and SA, whereas OMVs (a component of *C. sakazakii*) induced PVB, AVB, and SA. In agreement with these findings, *C. sakazakii* and its supernatant treatment did not show a substantial change in PR interval (only showed a non-significant trend), but OMV treatment significantly prolonged PR interval in mice.

In addition, *C. sakazakii*-derived OMVs can cross the placental barrier to induce arrhythmias in embryonic mice, which was consistent with the recent reports that bacterial OMVs have the ability to penetrate the intestinal epithelium and enter the bloodstream, where they can disseminate to various organs via the bloodstream.^25^ More importantly, maternal microbiota-derived OMVs have the potential to reach the intrauterine space and fetus during fetal development.^26^

We have found SCFAs employed as an effective antibacterial agent for *C. sakazakii*, but we did not further search for the antagonists or blockers of OMVs, which are directly involved in increase of arrhythmia induced by *C. sakazakii*. Although we demonstrated that lipids from OMVs trigger arrhythmia, we have not yet identified a candidate target for molecular therapy of sepsis-induced arrhythmia. Transcriptomic and lipidomic sequencing and analysis are required to further elucidate the detailed mechanism.

Sufficient evidence has suggested that several bacteria OMVs play important roles in sepsis progression, such as OMVs derived from *Escherichia coli*, which also directly led to sepsis-induced myocardial dysfunction.^27^^,^^28^^,^^29^^,^^30^ Therefore, we hypothesized that blocking OMVs could inhibit sepsis progression. And as expected, treatment of GW4869, a chemical inhibitor of exosome biogenesis, can effectively diminish the sepsis-induced cardiac inflammatory response, thereby attenuating myocardial depression and prolonged survival.^31^ The link between intestinal-flora-derived OMVs and arrhythmia needs further studied.

*Cronobacter* is considered an invasive opportunistic pathogen that can cause meningitis, necrotizing enterocolitis, and bacteremia or sepsis during infections in newborns and infants. The virulence of *Cronobacter* is thought to be related to a number of factors. It was observed that certain strains were capable of inducing diarrhea or causing notable ascites in newborn mice. The *Cronobacter* plasminogen activator gene-T6SS and fhaBC iron acquisition systems, along with a related adhesin locus, are carried by a family of RepFIB plasmids commonly associated with virulence in *C. sakazakii*. And its most representative virulence factors are outer membrane protein A. Furthermore, it has been reported that *Cronobacter* activates protein kinase C alpha (PKC-α) and inhibits mitogen-activated protein kinase (MAPK) through the phosphatidylinositol 3-kinase (PI3K)/Akt signaling pathway, ultimately invading cells. *Cronobacter* has the ability to evade the host immune response by utilizing immature dendritic cells that arise from the differentiation of monocytes, as well as macrophages.^32^

Studies have shown that due to their lipophilic nature, undissociated and uncharged organic acids are capable of crossing bacterial membranes. Once they enter bacteria with a more alkaline interior, the anions and protons of organic acids can lead to bacterial death by increasing osmotic pressure and disrupting the synthesis of biological macromolecules.^33^ The mechanism investigated in this study was that all kinds of SCFAs, including sodium acetate (C2), sodium propionate (C3), sodium butyrate (C4), succinic acid (C4-2), tartaric acid (C4-3), adipic acid (C6), exhibited excellent antimicrobial activity against *C. sakazakii* (a Gram-negative) *in vitro*. However, only sodium acetate and sodium butyrate exerted strong antiarrhythmic properties *in vivo*, suggesting that the antiarrhythmic effect of these compounds is not just dependent on their antibacterial activity. From a mechanistic perspective, the therapeutic effect of SCFAs in treating arrhythmias caused by sepsis is attributed to their antibacterial effect against *C. sakazakii*, but the involvement of SCFAs-related receptors, such as FFAR2 and FFAR3, also plays an important role. Increasing studies has shown that free fatty acid receptors (FFARs), especially FFAR2, have excellent protective effects against cardiovascular diseases, including diabetes, myocardial ischemia reperfusion, hypertension, arrhythmias, and so on.^19^^,^^34^^,^^35^ We found pretreatment by SCFAs, C2, C3, and C4 could increase the expression of FFAR2, whereas C3, C4, and C6 increased FFAR3 expression (Figure 8H). And, C2 and C4, but not C3 or C6, that preferentially activate FFAR2 reduced arrhythmogenesis (Figure 8B). FFAR3 was recently shown to promote cardiac inflammation/adverse remodeling that was opposed by RGS4,^36^ a GPCR signaling-regulating protein known to exert anti-arrhythmic effects.^37^ It is thus tempting to speculate that, at least, C3 (and/or C6) perhaps failed to reduce arrhythmogenesis (Figure 8B) and interleukin-6 (IL-6)/IL-1β/tumor necrosis factor alpha (TNF-α) levels (Figure 8G) because it activated the FFAR3. Simultaneously, we also examined the effect of 3-hydroxybutyrate on sepsis-induced arrhythmias. We found that 3-hydroxybutyrate has a good therapeutic effect on sepsis and also reduced the incidence of arrhythmias caused by sepsis. SCFAs reduced the incidence of arrhythmias from 50% to 16.67% within 12 h after sepsis, whereas 3-hydroxybutyrate only reduced the incidence of arrhythmias from 50% to 33.33%. In conclusion, although SCFAs show a better therapeutic effect, 3-hydroxybutyrate also has good application prospects. Recent studies have shown that 3-hydroxybutyrate can reduce sepsis-induced cardiac injury,^20^ which is consistent with our results.

Simultaneously, we also examined the effect of 3-hydroxybutyrate on sepsis-induced arrhythmias. We found that 3-hydroxybutyrate has a good therapeutic effect on sepsis and also helps reduce the incidence of arrhythmias caused by sepsis. SCFAs reduced the incidence of arrhythmias from 50% to 16.67%, whereas 3-hydroxybutyrate only reduced the incidence from 50% to 33.33% within 12 h after sepsis. In conclusion, although SCFAs show a better therapeutic effect, 3-hydroxybutyrate also has good application prospects. What’s more, recent studies have shown that 3-hydroxybutyrate can reduce sepsis-induced cardiac injury,^38^ which is consistent with our results. 3-Hydroxybutyrate can improve cardiovascular diseases, such as heart failure and myocardial hypertrophy, by reducing mitochondrial hyperacetylation and inflammation and regulating myocardial glucose-fatty acid metabolism.^39^^,^^40^^,^^41^

The antiarrhythmic effect of vitamin C in sepsis is not only dependent on its bacteriostatic effect on *C. sakazakii* but also attributed to other effects such as anti-inflammation, antioxidation, promoting angiogenesis, enhancing immunity, and epigenetic modifications.^42^

In conclusion, our study provided novel evidence of the interaction between cardiac electrophysiology and the microbiome in sepsis-induced arrhythmia. *C. sakazakii*-derived OMVs could promote arrhythmia by a disorder in ion channel protein expression. Short-chain fatty acids (SCFAs) exhibited good antibacterial activities on *C. sakazakii ex vivo*. Sodium acetate (C2) and sodium butyrate (C4) have reduced arrhythmia caused by *C. sakazakii* and sepsis *in vivo*, suggesting their potential as therapeutic strategy for sepsis. If these compounds exert therapeutic effects on arrhythmia in clinical practice and research in the foreseeable future, they could be particularly beneficial for cardiomyopathy patients.

### Limitations of the study

In conclusion, some limitations are still present in prevention and treatment of arrhythmias by killing *C. sakazakii*. Adipic acid increased arrhythmias and mortality in septic and Cs-treated mice, which was consistent with the toxicity of adipic acid in previous study.^43^ Succinate exacerbated arrhythmias in sepsis but reduced morbidity and mortality in sepsis. It is reported that succinate enhances muscle oxygen consumption and reduces reactive oxygen species (ROS) production in septic rats, consequently extending survival rates and optimizing metabolic profiles.^44^ Accumulated succinate in the septic heart plays a central role in accelerating heart damage, electrophysiological disorders, and diastolic and systolic dysfunction.^45^

## STAR★Methods

### Key resources table

**Table:** 

REAGENT or RESOURCE	SOURCE	IDENTIFIER
**Antibodies**		

anti-connexin 43/GJA1 rabbit antibody	Affinity	Cat# AF0137; RRID: AB_2833319
anti-connexin 40/GJA5 rabbit antibody	Affinity	Cat# DF13633; RRID: AB_2846652
anti-Nav1.5 rabbit antibody	Affinity	Cat# DF13217; RRID: AB_2846236
anti-Kv4.2/KCND2 rabbit antibody	Affinity	Cat# DF7675; RRID: AB_2841147
anti-occludin rabbit polyclonal antibody	HA	Cat# R1510-33; RRID: AB_3073341
anti-CACNA1C rabbit polyclonal antibody	HA	Cat# ER1803-49; RRID: AB_3069245
anti-Serca-2A recombinant rabbit antibody	HA	Cat# ET1703-01; RRID: AB_3070359
anti-ZO-1 rabbit antibody	Affinity	Cat# AF5145; RRID: AB_2837631
anti-IL-10 rabbit antibody	Affinity	Cat# DF6894; RRID: AB_2838853
anti-IL-1β rabbit antibody	Affinity	Cat# AF4006; RRID: AB_2801567
anti-TNF-α rabbit antibody	Affinity	Cat# AF7014; RRID: AB_2835319
anti-GPR43/FFAR2 rabbit antibody	Affinity	Cat# DF2746; RRID: AB_2839952
anti- GPR41/FFAR3 rabbit antibody	Affinity	Cat# AF9075; RRID: AB_2843266
anti-IL-6 mouse antibody	Affinity	Cat# DF6087; RRID: AB_2838055
anti-β actin rabbit antibody	Servicebio	Cat# GB11001; RRID: AB_2838055
anti-GAPDH mouse antibody	Servicebio	Cat# GB12002; RRID: AB_3206256

**Biological samples**		

CD-1 male mice	Huafukang Biotechnology Co., Ltd.	N/A
*Cronobacter. sakazakii*	Luwei Biotechnology Co., Ltd.	ATCC 29544

**Chemicals, peptides, and recombinant proteins**		

Sodium acetate	Picasso	127-09-3
Sodium propionate	Macklin	137-40-6
Sodium butyrate	Rhawn	156-54-7
Succinate	Rhawn	851916-42-2
Tartaric Acid	Rhawn	66749-60-8
Adipic acid	Rhawn	942037-55-0
3-Hydroxybutyrate	bidepharm	300-85-6
Vitamin C	Rhawn	26094-91-7
GLPG0974	Shyuanye	1391076-61-1
Sodium citrate	Beyotime	P0081
Agarose	Bidepharm	9012-36-6
4% paraformaldehyde	Beyotime	P0099
RIPA	Beyotime	P0013B
PMSF	Beyotime	ST506
Cardiac Troponin I (cTnI) Kit	Beijing Biolead biology technology CO, LTD.	Ctni-1-hs
Creatine kinase-MB (CK-MB) Kit	Wuhan Fine biology technology CO, LTD.	EM0929
Lactate dehydrogenase (LDH) Kit	Nanjing Jiancheng Bioengineering Institute,	A020
Alanine Aminotransferase (ALT) Kit	Nanjing Jiancheng Bioengineering Institute,	C009
Aspartate aminotransferase (AST) Kit	Nanjing Jiancheng Bioengineering Institute,	C010
Fecal DNA extraction Kit	Yeasen	18820ES70

### Resource availability

#### Lead contact

Further information and requests for resources and reagents should be directed to and will be fulfilled by the lead contact, Zhi-ping Fu (fzy1361351574@126.com).

#### Materials availability

This study did not generate new unique reagents.

#### Data and code availability

• PCR data reported in this paper will be shared by the lead contact upon reasonable request.
• This study did not generate new original data and code.
• Any additional information required to reanalyze the data reported in this paper is available from the lead contact upon request.

### Experimental model and study participant details

#### Animal

The Institutional Ethics Committee of North China University of Science and Technology reviewed and approved the study protocol with Approval No. 20230111, and the research was carried out in compliance with the principles formulated by "Animal Research: Reporting of *in Vivo* Experiments" (ARRIVE). Male CD-1 mice (8 weeks), were acquired from the Model Animal Research Center of North China University of Science and Technology. They were accommodated in a specific-pathogen-free (SPF) environment with a 12-hour light-dark cycle. The animals were randomly allocated to various groups, ensuring that each group had a similar number of mice and equal representation of all experimental conditions. The legends in the figures indicate the number of mice in each group, ensuring transparency and reproducibility of the research.

#### Sepsis model

Eight-week-old male mice were assigned to sham group and CLP group (n=10/ group). CLP surgery was employed to induce bacterial sepsis. Briefly, 100 mg/kg sodium pentobarbital (i.p.) was used to anesthetize mice before undergoing a 0.5-cm abdominal middle-line incision with the cecum exposed using a heat pad to keep warm. Next, we securely tied the cecum with 3-0 silk thread. Then, it was pierced twice using a 21-G needle, ensuring that the entire cecum was punctured close to its distal end. Gentle extrusion of stool through the puncture site followed by the closure of the abdomen was performed. The sham mice underwent the analogous operation, excluding the CLP step. Mice were given the CLP procedure for the purpose of assessing their health, morbidity, and survival rates for 10 days. Mice were divided into several treatment groups: a vehicle-treated group received distilled water (200 μL/ mouse), an SCFAs-treated group was administered a mixture containing 5% w/v all kind of SCFAs, a C2- treatment group received sodium acetate, a C3- treatment group received sodium propionate, a C4- treatment group received sodium butyrate, a C4-2- treatment group received succinate, a C4-3- treatment group received tartaric acid, a C6- treatment group received adipic acid, a 3-Hydroxybutyrate- treatment group received 3-Hydroxybutyrate, a Vc- treatment group received vitamin C, a GLPG0974-treatment group received GLPG0974. All treatment groups received their respective substances for 3 days before the CLP procedure. Each group comprised 6 animals. Given the acute severity of the disease, some spontaneous deaths occurred; however, ethical considerations precluded spontaneous death from being considered as an endpoint for this study. The animals were humanely euthanized when they displayed signs of morbidity. Following the treatment, the time at which each mouse passed away was documented for statistical analysis of mortality, assuming that its death occurred within the designated time frame. Upon completion of the designated time periods, all mice that remained alive were euthanized, and the samples were promptly gathered for examination. We scored all mice, detailed the specific scoring system for the parameters being observed, no symptoms (score 0); awkward gait, loose stools, some watery ocular discharge, fuzzy facial fur (score 1); hunched posture or slow gait, watery stools, some yellow ocular discharge, and a rough hair coat (score 2); Complete inability to move or lethargy, hemorrhagic diarrhea, red eyes accompanied by thick ocular discharge, and hair standing on end (score 3).

### Method details

#### Electrocardiograph

Eight-week-old mice were continuously anesthetized with inhalation of a 1-1.5% isoflurane-oxygen mixture (RWD, Batch). Surface electrocardiogram (ECG) was monitored by P3 plus (Data Sciences International) with subcutaneous platinum electrode was placed on lead Ⅱ. 20-min surface ECG samples of each animal were collected by LabChart 9 software. Heart rate, PR interval, R amplitude, and QRS intervals were measured from the 5-min mean curve. Ventricular tachycardia (VT) was defined as at least 4 consecutive premature ventricular beats (PVB).

#### Bacterial culture and antimicrobial activity

*C. sakazakii* (ATCC 29544) was cultivated in Luria-Bertani (LB) liquid media at 37°C. We test antimicrobial activity of SCFAs against *C. sakazakii*. *C. sakazakii* was cultured at 37°C overnight and centrifuged. The pellet was then resuspended in 1 mL liquid culture medium. The growth of bacteria was monitored by measuring the optical density (OD) at 600 nm using a visible spectrophotometer (JINGHUA 752, Shanghai, China) at different time point, and the bacteria growth curve was constructed based on these measurements. The inhibition zone diameter was measured using the punched hole with minor modifications. In brief, 100 μL of bacterial culture was evenly spread onto the LB solid medium. Then, a 0.9 mm diameter hole was punched, and 200 μL of distilled water or SCFAs solutions at concentrations of 10, 5, 1, 0.1, 0.01% were transferred into the holes, respectively. The diameter of the inhibition zone on each plate was observed and recorded. Finally, leakage of cytoplasm and nucleic acid was used to detected bacteria damage. Substances inside the cell leak out if the cell membrane breaks, such as DNA and RNA and proteins. The supernatant was filtered with disposable microporous membranes to remove the bacteria inside and measured for OD260 or 280 nm using an ultraviolet spectrophotometer (JINGHUA 752, Shanghai, China). Each drug was repeated three times.

#### Estimation of fecal- *C. sakazakii* abundance

To determine fecal *C. sakazakii* abundance, fecal DNA was extracted by a kit. DNA yield per gram of fecal weight was calculated and verified by PCR. PCR was used to amplify the RNA gene of *C. sakazakii* with the following cycling conditions: 95°C for 3 min, followed by 35 cycles of 94°C for 25 s, 55°C for 25 s, and 72°C for 10 s using primers (F: 5ʹ- CATATGGGTTTCGGTCATCGC-3ʹ, R: 5ʹ- GCTGATTTTCGATGAAGTGGACG-3ʹ), followed by a final extension at 72°C for 5 min. The 20 μL PCR reaction mixture, consisting of 10 ng template DNA and 0.8 μL of 10 μM primer, was prepared and the reactions were performed in triplicate. The 1.2% agarose gel was used to visualize the PCR products through electrophoresis. Subsequently, those bands with a size of approximately 186 bp were purified and analyzed using Image J.

#### Staining

Fresh heart, liver, spleen and other organs were excised, washed in PBS, and fixed in 4% paraformaldehyde. overnight at 4°C, then sliced 5-μm to cross sections. H&E and Sirius red staining were applied to the tissues. H&E was utilized to assess the architecture of the heart and other tissues. Sirius red was to measure the area of fibrosis.

#### Immunohistochemistry

After dewaxing and hydration, we treated the sections with hydrogen peroxide to eliminate endogenous peroxidase activity. We performed antigen retrieval using sodium citrate followed by blocking with goat serum. We incubated the sections with the primary antibody anti-Cx43 (1:100), and secondary antibody. Finally, specific markers and nuclei were stained, and the slides were observed and photographed.

#### Western blotting

Fresh heart and colon tissue was quickly removed from anesthetized mice and washed with ice-cold PBS; it was then dissolved by RIPA. Incubated the homogenates and centrifuged. The proteins were collected and quantified by kit. We heated the extracts at 96°C with an equal amount of Loading Buffer. To examine different protein levels, 20-60 μg of proteins were loaded and transferred onto a nitrocellulose (NC) filter or polyvinylidenefluoride (PVDF) membrane when proteins were separated electrophoretically. And then the membranes were blocked with non-fat milk and incubated with primary (1:2000) and secondary (1:2000) antibodies. The blots were detected using the ChemiDoc XRS+ Gel documentation system (Bio-Rad, Hercules, CA, USA).

#### Production, isolation and purification of OMVs

*C. sakazakii* were inoculated into sterilized LB and cultured overnight (37°C, 220 rpm). OMVs were collected by differential centrifugation. Briefly, the bacterial culture solution was centrifuged (4°C, 4000×g, 10 min) to obtain the supernatant. Purified OMVs were collected by ultracentrifugation using centrifugation (4°C, 200,000×g, 2 h). Its morphology and size were observed and captured by transmission electron microscopy (TEM) and nanoparticle tracking analysis (NAT), respectively. The OMVs were resuspended in PBS and orally gavaged to mice at a daily dose of 20 μg per mouse.

#### Transmission electron microscopy

Purified *C. sakazakii*-derived OMVs pellet obtained during OMVs isolation was resuspended in glutaraldehyde solution and placed on a 400 mesh Formvarcoated copper microscopy grid. The grid was fixed and stained by 1% aqueous uranyl acetate. Photographs of the sections were taken using a JEM-201003040701 electron microscope (JEOL, Tokyo, Japan).

#### Nanoparticle tracking analysis (NTA)

The Zetaview system was utilized for nanoparticle tracking analysis (NTA) to measure the quantity of OMV particles. OMVs were diluted by 1:300 in filtered autoclaved phosphate-buffered (PB) solutions and loaded onto Nano Sight LM10 sizer and counter (Malvern Nano Sight LM10, Malvern, UK). For each technical replicate, the particle size and quantity were recorded for 1 minute. Each sample was analyzed with 10 technical replicates and 3 biological replicates.

#### Measurements of AST, ALT, LDH, CK-MB and cTnI activities

The myocardial enzyme spectrums, hepatic function of mice were analyzed by blood biochemical analysis. We detect the levels of cardiac Troponin I (cTnI), creatine kinase-MB (CK-MB) and lactate dehydrogenase (LDH) in serum, and alanine aminotransferase (ALT) and aspartate aminotransferase (AST) in plasma according to the reagent and the biochemical kit's instructions, and the PBS treated mice were used as the control group.

### Quantification and statistical analysis

Data are presented as the mean ± standard deviation. χ2 test or Fisher's exact test were used to evaluate differences between percentages. Differences between two groups were determined using Student's non-paired t-test. The groups with normally distributed parametric data were tested using one-way analysis of variance (ANOVA), followed by Tukey's (for the same number of samples) or Sidak's (for different numbers of samples) multiple comparison test (for more than two groups). Non-parametric data were compared using the Mann-Whitney's U test, while differences between groups at multiple time points were analyzed using two-way ANOVA followed by Tukey's multiple comparison test. Variables with *P* < 0.05 at univariable analysis were then included as covariates in multivariable analysis. The Kaplan–Meier method and Log-rank (MantelCox) test were used to construct and determine the survival rate between all treatment groups, respectively. And, n represents number of animals. Prism 9 software (GraphPad Prism Software Inc, San Diego, CA) and SPSS 18 (SPSS, Inc, IL, USA) were utilized for all statistical analyses. Statistical significance was only considered for differences with a *P*-value of less than 0.05.
