# Supplementary material for: Cronobacter sakazakii induced sepsis-associated arrhythmias through its outer membrane vesicles
Source: iScience. 2024 Jul 25;27(9):110572. doi: 10.1016/j.isci.2024.110572 (PMC11369384; doi:10.1016/j.isci.2024.110572)
Supplement: Document S1. Figure S1 [file mmc1.pdf]

iScience, Volume *27*

Supplemental information

Cronobacter sakazakii induced sepsis-associated arrhythmias through its outer membrane vesicles

Zhi-ping Fu, Shuang Lee, Rui-yao Wang, and Yu-qing Wang

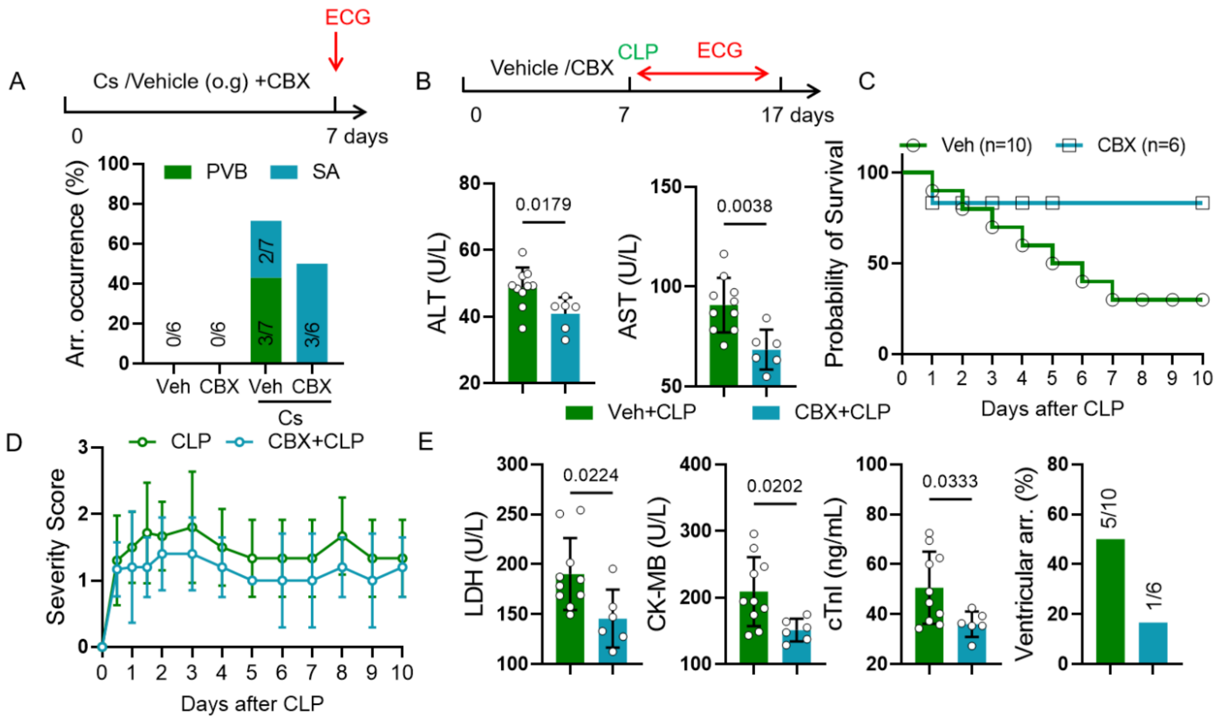

Supplementary figure 1. Carbenoxolone reduced arrhythmia caused by *C.sakazakii* and CLP surgery, related to figure 2..

A.) Experimental design for *C. sakazakii* and carbenoxolone (CBX) treatment and ECG testing. Summary the occurrence of ventricular arrhythmia (VA) in *C. sakazaki*i gavaged mice that pretreated with CBX. Numbers in parentheses indicate the number of mice that occurred VA after *C. sakazakii* treated.

B.) Experimental design for CLP model, CBX treatment, and surface ECG testing. Serum ALT, AST levels were quantified using commercial assay kits. Data are presented as the mean ± SD. Data analyzed by unpaired two tailed Student's t test.

C., D.) Probability of survival and sepsis severity score were calculated for each group. Data are presented as the mean ± SD.

E.) Cardiac LDH, CK-MB, cTnI levels were quantified using commercial assay kits. The occurrence of ventricular arrhythmia (VA) in CLP mice pretreated with CBX. Numbers in parentheses indicate the number of mice that occurred VA in septic mice after CBX treated. Data are presented as the mean ± SD. Data analyzed by unpaired two tailed Student's t test.
